# Peer-to-peer health promotion interventions among African American men: a scoping review protocol

**DOI:** 10.1186/s13643-021-01737-y

**Published:** 2021-06-22

**Authors:** Guillermo M. Wippold, Sarah Grace Frary, Demetrius Abshire, Dawn K. Wilson

**Affiliations:** 1 Department of Psychology, University of South Carolina, 1512 Pendleton Avenue, Barnwell College, Mailbox 38, Columbia, South Carolina 29208 USA; 2 College of Nursing, University of South Carolina, Columbia, USA

**Keywords:** African American, Men, Health promotion, Disease prevention, Quality of life, Intervention, Peer-based

## Abstract

**Background:**

Health promotion efforts among African American men have been met with significant challenges and have produced limited results. Interventions that do not align with the values, perspectives, and preferences of African American men often produce less effective results. Research among African American men has provided compelling evidence that these men prefer informal networks of health support. Recent successful health promotion efforts among these men have benefited from peer-to-peer models of implementation. To date, no known scoping or systematic review of peer-to-peer health promotion interventions among African American men has been conducted. The goal of this scoping review is to understand the extent of, design, implementation, and use of peer-to-peer interventions to promote health, improve quality of life, and prevent disease among African American men.

**Methods:**

A review of the literature will be performed in PubMED, EMBASE, PsycInfo, CINAHL, and Web of Science. The development of this protocol was guided by the work of Arksey and O’Malley and the PICOS statement. Reporting will be guided by the PRISMA-ScR checklist. Eligible studies include those testing the effects of a peer-to-peer health promotion intervention targeting African American men. A comparison group will not be required. For the purposes of the current review, “peers” will be limited to other African American men. An initial screening of the titles and abstracts of potentially eligible studies will be completed by two independent reviewers. The full text of records that appear to meet the eligibility criteria will be accessed and further screened. Data will then be extracted and collected using a custom Microsoft Excel spreadsheet. Extracted data will include authors’ name and publication year, target health issue(s), design of the intervention, components of the intervention, peer-led components of the intervention, peer role, length and type of training for peer leaders, intervention duration, frequency of the intervention, study design and number of participants, and main outcomes. Finally, results will be presented in table format and summarized in text format.

**Discussion:**

Results will have implications for the design, implementation, and evaluation of health promotion interventions among African American men.

**Systematic review registration:**

PROSPERO CRD42020198664

**Supplementary Information:**

The online version contains supplementary material available at 10.1186/s13643-021-01737-y.

## Background

African American men experience high rates of adverse health indicators that contribute to a lower life expectancy among these men than any other racial-gender group in the USA [1, 2]. The contributions of these health indicators to life expectancy are worrisome because health promotion efforts targeting African American men have produced limited results and are met with certain difficulties. For example, recruiting and retaining these men in health promotion efforts can pose a significant challenge [3–6]. When recruitment and retention efforts are successful, health promotion efforts targeting individuals from underserved communities are often hindered by limited cultural sensitivity and tailoring [7, 8]—evidence-based health promotion interventions that do not align with the values, perspectives, and preferences of the target community often produce less effective results for these communities [9–11].

Effective health promotion efforts to address high rates of adverse health indicators among African American men need to be culturally responsive [12]. There is strong support derived from research involving African American men that indicates these men prefer informal networks of support [13], such as family, friends, and other forms of kinship [14–19]. This preference for informal networks of support has led to the development of health promotion interventions delivered in barbershops, which is a community focal point for African American men [20–26]. In addition to receiving a haircut, African American men use barbershops as a sanctuary to dialogue with other African American men [27]—they are often perceived as safe places for difficult conversations, such as conversations about sensitive health topics. Health promotion efforts conducted at the barbershop benefit from established networks of trust. These interventions are often successful in recruiting and retaining African American men and deliver content in manners that align with the values, perspectives, and preferences of these men largely because they are implemented using a peer-to-peer model.

Peer-to-peer models of health promotion capitalize on social and established networks of trust [28]. These models of health promotion can readily be adapted to align with the values, perspectives, and preferences of the target community. Furthermore, these models are sustainable, scalable, and cost effective [29]—they have the potential to promote health equity among communities experiencing health disparities [30]. Despite the apparent benefits of peer-to-peer models of health promotion among African American men, there have been no known scoping or systematic reviews conducted to understand the use of peer-to-peer interventions to promote health among African American men. To the authors of this protocol’s knowledge, there is one known protocol for a systematic review of health promotion interventions for African Americans delivered in barbershops and salons and two completed systematic reviews reviewing the use of peer-based health promotion interventions [30–32]. The protocol for the known systematic review of interventions conducted in barbershops and salons consists of interventions that target both men and women [31]. Additionally, the protocol focuses on location (i.e., the barbershop), rather than the method (i.e., peer-to-peer model) of delivery for the health promotion effort [31]. The two known completed systematic reviews provide mixed evidence for the use of peer-to-peer models as a method, though were not restricted to literature examining health promotion efforts specifically targeting African American men [30, 32].

### Objectives of the current review

There is an urgent need to develop innovative health promotion programs for African American men [33]—these men have the shortest life expectancy of any racial-gender group in the USA and billions of dollars per year in excess medical costs are expended due to health disparities [2, 34]. It is known that “one-size-fits-all” efforts have produced limited results [35]. Therefore, health research that focuses solely on health promotion among African American men will help in understanding the foundations of successful health promotion efforts among these men [33]. This scoping review is informed by an overwhelming amount of evidence suggesting that African American men prefer and benefit from informal networks of social support and that health promotion efforts can benefit from leveraging these informal networks [13–19]. This scoping review is the first known review with the objectives to understand the design, implementation, and benefits of peer-to-peer models of health promotion and disease prevention among African American men.

## Methods

The design for this scoping review was planned by the authors to inform the objectives by addressing the following research questions:

1. What peer-to-peer health promotion, quality of life improvement, and disease prevention interventions among African American men have been conducted?
2. How were these interventions designed (i.e., with or without community input, duration, theoretical orientation) and implemented (i.e., components, recruitment strategy, delivery of intervention)?
3. What were the roles of the peer leaders in these interventions?
4. How were the peer leaders trained?
5. To what degree were the interventions peer-to-peer (e.g., fully peer-to-peer implemented, partially peer-to-peer implemented)?
6. What were the settings (e.g., telephone, church, workplace) of these interventions?
7. What health outcomes were targeted by these interventions?
8. What are the effects of the interventions on the targeted health outcomes?

The scoping review protocol was informed by the methodological criteria for scoping reviews set forth by Arksey and O’Malley [36]—we have already identified the research questions (see above) and will next identify relevant studies, select those studies, chart the data, and summarize and report the results [37]. The reporting of this scoping review will follow the reporting guidelines and criteria set in the Preferred Reporting Items for Systematic Reviews and Meta-Analyses Extension for Scoping Reviews (PRISMA-ScR) [38]. See supplementary file for the Preferred Reporting Items for Systematic Review and Meta-Analysis Extension for Scoping Reviews (PRISMA-ScR) checklist [39] for an aggregate of relevant data and indicator of reporting transparency.

### Eligibility criteria

Studies included in the proposed scoping review will be restricted to studies examining the impact of peer-implemented interventions (*I*ntervention) seeking to improve one or more health indicators (*O*utcome) among African American men (age 18 or above; *P*opulation). Given the limited amount of documented health promotion efforts targeting this population, interventions and feasibility trials using single-group pre-post study, post-test only study, non-randomized controlled trial, and randomized controlled trial (RCT) study designs will be included (*S*tudy Type). Comparison groups will not be required (*C*omparison). See Table 1. Thus, studies will be included if they (1) target African American men (age 18 or above), (2) seek to improve one or more health outcomes, and (3) include at least one peer-to-peer component. Studies will be excluded if they (1) target African American men and women, (2) target African American men and men of other race/ethnicities, and (3) target adults and children. The rationale for our exclusion criteria was guided by experts in health promotion among African American men who suggest that focusing exclusively on these men facilitates the identification of unique pathways impacting their health, in addition to mechanisms that can serve as the foundation of health promotion efforts [33, 40, 41]. Excluding studies that include African American women or men from other racial/ethnic groups was therefore deemed important for ensuring that interventions were specifically designed to address the unique considerations of African American men. Although some authors may report findings specific to African American men, our focus is on how interventions are customized for this population. Given the limited amount of health promotion interventions conducted among African American men, this review will not be restricted to studies published before, on, or after a predetermined year. Only articles written in English will be included because interventions tailored for African American men were presumed to be conducted within the USA and published in an American English publishing outlet.

**Table 1:** PICOS statement table

Element	Description
Population	African American men above the age of 18
Intervention	Peer-implemented health promotion interventions
Comparison	Comparison groups will not be required
Outcome	One or more health indicators
Study Type	Interventions and feasibility trials using single-group pre-post study, post-test only study, non-randomized controlled trial, and randomized controlled trial

The following definitions for peer and peer-to-peer interventions will be used to determine eligibility:

1. *Peers* are individuals who share key personal characteristics, circumstances, or experiences with a population [42]. Although one can be a peer based on sexual orientation, occupation, age, or sharing a common experience (e.g., veterans of war), the proposed study will be restricted to individuals who are peers based on gender and race (i.e., African American males)—categories referred to as “master categories” [42]. In the health promotion literature, the term “peers” often refer to trained individuals [43]. The present study will define peers as trained or untrained (e.g., lay health advisors) African American men.
2. *Peer-to-peer interventions*, or peer-based interventions, are interventions implemented entirely or in part by peers. In the context of the proposed scoping review, peer-to-peer interventions are interventions led, though not necessarily designed, by African American men that seek to promote an aspect of physical, psychological, or social functioning among other African American men.

### Data sources

Information will be identified by searching the following bibliographic databases: PubMED, EMBASE (Ovid), PsycInfo (Ebsco), CINAHL (Ebsco), and Web of Science (Clarivate). Grey literature databases and repositories will not be searched given concerns about the methodological rigor (methodologies are a primary focus of this review) of grey literature interventions. Additionally, many interventions found in the grey literature were not assessed for ethics nor approved by an institutional review board—a salient concern given the population of interest (i.e., African American males) and the long history of ethical violations experienced by these men that were conducted by health researchers.

### Search strategy

The literature search strategy was developed by the PI in consultation with librarians at the University of South Carolina. The keyword *peer* or any variation of that keyword will not be included in the search strategy because it is often not explicitly mentioned in the text though can be readily identified in the Methods or description of the intervention. The PI designed the search strategy that will be used for the proposed scoping review, which will include keywords such as intervention, African American, Black, Male, and health promotion. Complete search terms to be used within the search are included in the Supplementary Materials section.

### Literature screening and selection

Titles and abstracts yielded by the search strategy will be uploaded to EndNote by GMW and duplicates will be removed. Once duplicates are removed, GMW will upload the remaining titles and abstracts to Rayyan—a reference managing software that allows for organization of research articles and collaboration among investigators [44]. GMW and SGF will each independently screen all titles and abstracts against the eligibility criteria to identify potentially relevant studies. GMW and SGF will then access full-text copies of the included titles and screen against the eligibility criteria. Any disagreements will be resolved by DA.

### Data extraction

A piloted data extraction form was designed for data collection. The extracted data will be collected by using Microsoft Excel. The extracted data from potential studies will include items such as authors’ name and publication year, target health issue(s), design of the intervention (i.e., with or without target community input), components of the intervention (e.g., information session), peer-led components of the intervention (e.g., all components were peer-led or certain and which components were peer-led), peer role, length and type of training for peer leaders, intervention duration, frequency of the intervention, setting of the intervention, study design and number of participants, and main outcomes. Peer roles will be assessed using the categories developed by Ramchand et al. [32]. See Table 2 for a summary of the categories. If the desired extracted data is not presented in the published study, GMW will email the authors of the study to request the information.

**Table 2:** Peer roles as described by Ramchand et al. [32]

Role	Description of role
Peer counselor	A peer who provided knowledge, guidance, and concrete tools
Peer educator	A peer who delivered formal education or training utilizing a protocolized curriculum and approach, and not involving a therapeutic relationship
Peer support	Informal and unstructured support to individuals such as providing reminders, encouragement or reinforcement, informal coaching, and sharing personal experiences or narrative, often as a “buddy” or “partner” in the intervention
Peer facilitator	A peer responsible for facilitating group interactions with the primary purpose of creating or strengthening relationships between and among individuals to help them set and reach goals together
Peer case manager	A peer who helped other access or coordinate health and social services including referring the participant to resources, or managing their activities within the intervention

### Data synthesis

Extracted data will be summarized by GMW and SGF. The 2020 version of the Joanna Briggs Institute Reviewer’s Manual will be used to guide data syntheses [45]. A table created by GMW and SGF will include results of the extracted information. This table will be accompanied by a descriptive summary, which will describe how the results of the review may inform the future development of peer-to-peer health promotion interventions among African American men. DKW is an expert on developing and implementing innovative, theoretically based health promotion interventions among African Americans. GMW and DA have experience developing and implementing health promotion interventions among African Americans. The PRISMA-ScR guidelines will also be used as a guide to report our findings [38].

## Discussion

There is an urgent need to improve the health of African American men. Many health promotion efforts targeting these men have been limited by recruitment and retention [3–6], in addition to a lack of cultural sensitivity and tailoring [7, 8]. One notable exception consists of barbershop-based interventions. These interventions are often successful in recruiting and retaining these men, are culturally sensitive and tailored, and produce significant health improvements among African American men [20–25]. It is likely that the design and method of delivery (i.e., peer-to-peer) rather than location (i.e., barbershop) produce these significant health improvements. Despite the likely benefit of the design and method of delivery, no known scoping review, systematic review, or meta-analysis has been conducted to examine the use of peer-to-peer strategies to promote health among African American men—an approach that is congruent with research findings that indicate that these men prefer and benefit from informal networks of social support [13–19].

The results of this novel scoping review will describe the development, implementation, and benefits of peer-to-peer health promotion interventions among African American men. Specific data on the design of the intervention (i.e., with or without target community input), components of the intervention (e.g., information session), peer-led components of the intervention (e.g., all components were peer-led or certain and which components were peer-led), peer role, length and type of training for peer leaders, intervention duration, frequency of the intervention, setting of the intervention, study design and number of participants, and main outcomes will be acquired. Such data and the results drawn from these data will be valuable to a wide range of health promotion specialists beyond those specialists conducting barbershop-based interventions. Peer-to-peer models can be implemented by health promotion specialists using lay health advisors and community health workers. Additionally, these results will be of value to policy-makers interested in promoting the health of an at-risk and underserved community—national and international funding agencies (e.g., World Health Organization, Patient-Centered Outcomes Research Institute, National Institutes of Health) have highlighted the importance of community-informed and peer-based health promotion. Finally, the results of the present scoping review will also support the design and implementation of future systematic reviews and meta-analyses examining specific health promotion strategies among African American men.

## Supplementary Information

**Additional file 1.** Preferred Reporting Items for Systematic reviews and Meta-Analyses extension for Scoping Reviews (PRISMA-ScR) checklist.

**Additional file 2.** Supplementary Materials. Search Strategy.

## Data Availability

All data generated during this study will be included in the published article. Results will be widely disseminated in the form of peer-reviewed articles and conference presentations.

## Abbreviations

PRISMA-ScR: Preferred Reporting Items for Systematic Reviews and Meta-Analyses Extension for Scoping Reviews
PRISMA-P: Preferred Reporting Items for Systematic Review and Meta-Analysis Protocols
PROSPERO: The International Prospective Register of Systematic Reviews
PICOS: *P*opulation, *I*ntervention, *C*omparison, *O*utcome, and *S*tudy Type
RCT: Randomized controlled trial
EMBASE: Excerpta Medica database
CINAHL: Cumulative Index to Nursing and Allied Health Literature
PI: Primary investigator
