# Supplementary material for: Peer-to-peer health promotion interventions among African American men: a scoping review protocol
Source: Syst Rev. 2021 Jun 22;10:184. doi: 10.1186/s13643-021-01737-y (PMC8218504; doi:10.1186/s13643-021-01737-y)
Supplement: Supplementary file 2 — Additional file 2. Supplementary Materials. Search Strategy. [file 13643_2021_1737_MOESM2_ESM.docx]

**Supplementary Materials**

**Search Strategy**

PubMED:

((((intervent*[Title] OR rehabilit*[Title] OR prevention[Title] OR program[Title] OR improv*[Title] OR counsel*[Title] OR randomized control* trial*[Title] OR RCT[Title] OR barbershop[Title] OR outcome*[Title]) AND (African American*[Title/Abstract] OR black[Title/Abstract] OR blacks[Title/Abstract])) AND (Male[Title/Abstract] OR males[Title/Abstract] OR men[Title/Abstract] OR adult*[Title/Abstract])) AND (health promotion[Title/Abstract] OR health behavior[Title/Abstract] OR health attitudes[Title/Abstract] OR health education[Title/Abstract] OR health knowledge[Title/Abstract] OR lifestyle[Title/Abstract] OR health literacy[Title/Abstract] OR diabetes[Title/Abstract])) NOT (female[Title/Abstract] OR women[Title/Abstract] OR woman[Title/Abstract] OR girl*[Title/Abstract] OR adolescen*[Title/Abstract] OR teen*[Title/Abstract])

EMBASE:

(intervent*:ti OR rehabilit*:ti OR prevention:ti OR program:ti OR improv*:ti OR counsel*:ti OR 'randomized control* trial*':ti OR rct:ti OR barbershop:ti OR outcome*:ti) AND ('african american*':ab,ti OR black:ab,ti OR blacks:ab,ti) AND (male:ab,ti OR males:ab,ti OR men:ab,ti OR adult*:ab,ti) AND ('health promotion':ab,ti OR 'health behavior':ab,ti OR 'health attitudes':ab,ti OR 'health education':ab,ti OR 'health knowledge':ab,ti OR lifestyle:ab,ti OR 'health literacy':ab,ti OR diabetes:ab,ti) NOT (female:ab,ti OR women:ab,ti OR woman:ab,ti OR girl*:ab,ti OR adolescen*:ab,ti OR teen*:ab,ti)

PsycInfo:

TI ( intervent* OR rehabilit* OR prevention OR program OR improv* OR counsel* OR randomized control* trial* OR RCT OR barbershop OR outcome* ) AND AB ( "African American*" OR black OR blacks ) AND AB ( Male OR males OR men OR adult* ) AND AB ( "health promotion" OR "health behavior" OR "health attitudes" OR "health education" OR "health knowledge" OR lifestyle OR "health literacy" OR diabetes ) NOT AB ( female OR women OR woman OR girl* OR adolescen* OR teen* )

CINAHL:

TI ( intervent* OR rehabilit* OR prevention OR program OR improv* OR counsel* OR randomized control* trial* OR RCT OR barbershop OR outcome* ) AND AB ( "African American*" OR black OR blacks ) AND AB ( Male OR males OR men OR adult* ) AND AB ( "health promotion" OR "health behavior" OR "health attitudes" OR "health education" OR "health knowledge" OR lifestyle OR "health literacy" OR diabetes ) NOT AB ( female OR women OR woman OR girl* OR adolescen* OR teen* )

Web of Science:

**TITLE:**(intervent*  OR rehabilit*  OR prevention  OR program  OR improv*  OR counsel*  OR randomized control*trial*  OR RCT  OR barbershop  OR outcome*) *AND* **TOPIC:** ("African American*"  OR black  OR blacks) *AND* **TOPIC:** (Male  OR males  OR men  OR adult*) *AND* **TOPIC:** ("health promotion"  OR "health behavior"  OR "health attitudes"  OR "health education"  OR "health knowledge"  OR lifestyle  OR "health literacy"  OR diabetes) *NOT* **TOPIC:** (female  OR women  OR woman  OR girl*  OR adolescen*  OR teen*)
